# Urban mobility trends and climate change: sustainability policies in the parking industry

**DOI:** 10.1007/s11356-023-26925-2

**Published:** 2023-05-01

**Authors:** Raquel Fernández-González, Félix Puime-Guillén, Victor Manuel Ferreira Moutinho, Helena Maria Santos de Oliveira

**Affiliations:** 1 Department of Applied Economics, ERENEA-ECOBAS, University of Vigo, Lagoas-Marcosende S/N, 36310 Vigo, Spain; 2 Department of Business, University of A Coruña, 15008 A Coruña, Spain; 3 Management and Economics Department and NECE-UBI, University of Beira Interior, Rua Marquês d’Ávila E Bolama, 6201-001 Covilha, Portugal; 4 Accounting and Management Department and CEOS.PP, Polytechnic Porto, School of Accounting and Administration of Porto and NECE-UBI, University of Beira Interior, Covilha, Portugal

**Keywords:** Parking, Cleaner production, Urban mobility, Sustainability policies; Green economy, Spain

## Abstract

The concern to create cleaner and more ecosystem-friendly production processes has extended to the parking sector in Spain. Since the creation of the multi-level institutional framework for sustainable mobility management (mainly composed of the Infrastructure, Transport and Housing Plan 2012–2024, the Sustainable Urban Mobility Plans, Law 9/2006, and Law 9/2017), environmental considerations, including sustainable management certificates, have occupied a privileged place in public procedures for the management of parking structures and regulated surface parking facilities. Although there have been previous academic studies on the design and implementation of SUMPs and the growth of the parking sector, this article is novel in that it analyzes the market concentration of the parking sector in a scenario where climate change policies are crucial and the importance of sustainability certificates takes on a new meaning. Therefore, the objective of this article is to analyze whether the growing importance of environmental aspects has led to an increase in the concentration level of the parking sector in Spain. For this purpose, several concentration and stability indices are calculated. The results show that, although there are additional factors, the certification of a cleaner activity is relevant in the process of public tenders in the sector, which has served to strengthen the dominance of the most prominent companies in the sector that are in possession of environmental certificates. This shows that environmental policies can also have negative effects on the market, so the results of this analysis are of great value to policymakers.

## Introduction

Through the Sustainable Development Goals (SDGs) agenda, the United Nations has created a universal framework to promote economic growth and social improvement while pursuing environmental sustainability. These goals have been agreed upon by developed and emerging countries, with a commitment to achieve them (Anwar et al. 2022). Despite the fact that the implementation and design of policies related to the SDGs are at different speeds, this agenda represents a global effort to address climate change. This goal is implicitly included in SDG 13, which focuses on the capacity to create shared knowledge, establish plans, and promote mechanisms to manage climate change (Guang-Wen et al. 2022).

Since 1992, the United Nations has also supported the Conference of the Parties (COP). These conferences provide a global forum for exchanging ideas, proposing and agreeing on effective measures to combat climate change. At the recent COP26 in Glasgow, Scotland, efforts focused on reaching an agreement to reduce emissions of polluting gases into the atmosphere and to limit the increase in global temperature to 1.5 °C above pre-industrial levels (Murshed et al. 2022). The agreements reached focused on reducing methane emissions by 30% by 2030, reducing deforestation, and reducing CO_2_ emissions by 45% by 2030 (Ali et al. 2022). These initiatives follow a long list of policies that have been implemented for more than three decades, with mixed results (Shakib et al. 2022).

For all these reasons, the main efforts of the environmental science community in the last decade have been focused on mitigating climate change (Caballero-Miguez and Fernández-González 2015; Howarth et al. 2020; Jakučionytė-Skodienė and Liobikienė 2021). In its fifth report, the Intergovernmental Panel on Climate Change (IPCC) recommended that the global mean temperature should not exceed 2 °C compared to the pre-industrial climate record (Knutti et al. 2016; Tamaki et al. 2017). Thus, temperature variability has been analyzed in numerous studies (Braganza et al. 2004; Gasparrini et al. 2017; Hertig and Jacobeit 2008; Howe et al. 2013; Loarie et al. 2009). Overall, one of the main contributors to global overheating is transportation (Álvarez-Díaz et al. 2017; Hickman and Banister 2014; Santos 2017).

Globally, the transportation sector is responsible for 23% of CO_2_ emissions and 14% of other greenhouse gases (GHGs), with road transport as the largest exponent (BP 2017). These emissions are a consequence of the widespread use of internal combustion engines (ICEs), as 95% of road transport uses this type of engine, whose energy source is fossil fuels (Kalghatgi 2018; US EPA 2016). The 1.2 billion private cars and 380 million commercial vehicles currently on the world’s roads are a constant source of CO_2_ from burning fossil fuels (Bilgin et al. 2015; Kalghatgi 2018). In addition, global CO_2_ emissions from transportation use are projected to double by 2050 (Higham and Font 2020).

Faced with this scenario, the European Union (EU), one of the main supranational organizations in the fight against climate change, has encouraged its member countries to design and implement active policies against pollution caused by road transport, which is responsible for 27% of CO_2_ emissions in the EU (Gössling et al. 2016; Bart 2010; Tsakalidis et al. 2020). Spain, a member of the EU, has created an institutional framework organized at different levels, whose national basis, the Infrastructure, Transport and Housing Plan 2012–2024 (PITVI 2012–2024), has been complemented by Sustainable Urban Mobility Plans (SUMPs) at the local level since 2014 (Mozos-Blanco et al. 2018; Naranjo Gómez et al. 2020).

Parking promotion policies in road transport have been of great importance in mobility sustainability policies in Spain (Antolín et al. 2019; Braulio-Gonzalo et al. 2015). Dissuasion parking facilities, the implementation of parking with a fractional payment system in urban areas, the creation of intermodal parking lots, or the establishment of administrative concession parking lots have been widespread measures in SUMPs (Ministerio de Fomento 2013). As a result of this policy, the number of parking spaces in Spain has tripled in 9 years, between 2010 (480,000 spaces) and 2019 (1,500,000 spaces) (DBK 2011, 2021). The high volume of turnover, 2000 million euros per year in Spain, and the policies of consolidation and promotion of this sector attracted numerous companies in the last decade (Expansión 2021).

The objective of this article is to analyze the degree of concentration and stability of the parking market in Spain, following the promotion of sustainability and urban mobility policies. Although several articles have analyzed the parking sector in Spain, these studies have mostly focused on technical aspects (Arellanos-Verdejo et al. 2021; Gomez-Ullate et al. 2011; Sotres et al. 2019; Vlahogianni et al. 2016), microeconomic (Caicedo et al. 2006; Ibeas et al. 2014), or have based their analysis on the link between mobility and tourism (Blázquez-Salom et al. 2021; Santana-Santana et al. 2020; Snider et al. 2015). Therefore, this article presents an analysis of the market from a methodology that has not yet been applied in an academic way to this sector.

Sustainability is an increasingly sought-after attribute in all sectors of the economy. The parking sector, directly related to mobility and transport, is closely linked to sustainable production. The very existence of parking facilities, especially park and ride facilities, is a commitment to reduce emissions of polluting gases. However, the management of parking facilities must also be sustainable, especially considering that this is a desirable social value. The relationship between the parking sector and the public administration is close, since part of the activity of this sector depends on public contracts where sustainability is a criterion to be followed. For this reason, the possession of environmental and sustainable management certificates gives the parking company that holds them a comparative advantage over its competitors when public contracts are awarded. The purpose of this analysis is to examine whether the most sustainable companies, which also possess environmental certificates, are also the ones that dominate the market, since they monopolize a large part of the public contracts.

## The institutional framework of the parking sector in Spain

**Fig. 1:**
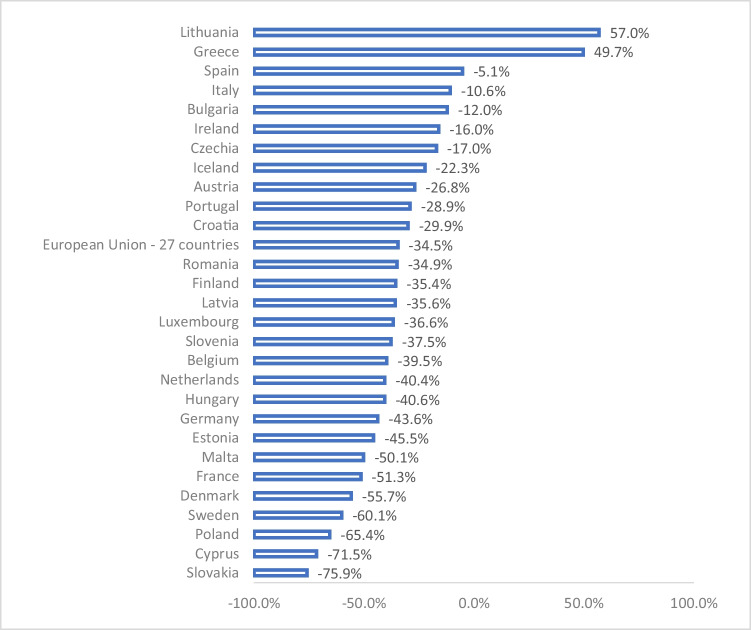
Percentage change in total greenhouse gas emissions from transport in Europe (2010–2021). Source: own elaboration based on European Environment Agency (2021)

During the twentieth century, characterized as the era of individual and private transportation, various measures were developed to respond to the growing demand for motorized mobility (Schafer 1998). These policies have evolved into the current trend of promoting sustainable mobility management (Kim et al. 2013). In 2007, within the framework of the European Conference of Ministers of Transport, the adoption of policies to reduce greenhouse gas emissions was promoted as a priority mobility objective in the European Union, with special attention to those countries with a negative pollution trend (Hensher 2008). Spain was included in this group of countries because it is one of the five European countries with the lowest rate of reduction of greenhouse gas emissions from transport between 2010 and 2021 (Fig. 1) (European Environment Agency 2021).

**Fig. 2:**
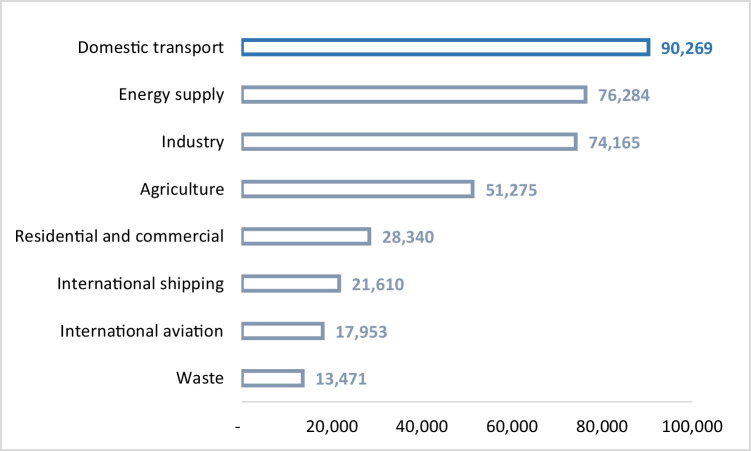
Emission of greenhouse gas emissions by sector in Spain in 2018 (kt CO2 eq). Source: own elaboration based on European Environment Agency (2021)

**Fig. 3:**
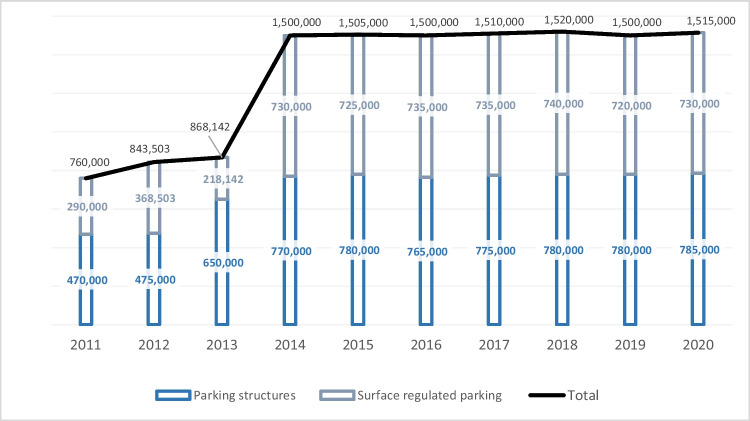
Number of parking spaces by type and year in Spain. Source: own elaboration based on Asesga (2021) and DBK Observatorio Sectorial (2021)

As shown in Fig. 2, the transport sector is the largest contributor to greenhouse gas emissions in Spain (Navas-Anguita et al. 2018). In particular, domestic transport, which is mainly composed of road transport, ranks first in terms of emissions (24%). Consequently, in the context of the EU plan to reduce greenhouse gas emissions, Spanish mobility policies related to the environmental impact of road transport have proliferated with the approval of the PITVI 2012–2024 (national level) and the SUMPs (regional, supra-municipal, or municipal level) (BOE 2011a). Both types of regulations include parking management as a key measure to reduce the proportion of vehicles circulating on urban streets. Specifically, among other measures, they promote deterrent parking, which facilitates intermodal switching to other modes of transport, and urban blue zone parking, which is paid and even temporarily restricted (Laconte 2018; Yashiro and Kato 2019). As a result, the number of parking spaces in both structured and regulated surface parking lots has increased in Spain over the past decade (Fig. 3).

### Sustainable mobility management plans

It is necessary to emphasize that the sustainable urban mobility policy in Spain is implemented through multilevel governance (Geels 2014; Moradi and Vagnoni 2018). It is a “top-down” governance process. The supranational agent (the European Union) is the one that determines the guidelines for the institutional change towards integrated and sustainable mobility, and both the national agent (the Spanish State) and the regional/local agent (Spanish regions or localities) design the corresponding policies, following the evolution of the rules of the game that determine the incentives of the higher-level agents.

For example, the Spanish Infrastructure, Transport and Housing Plan 2012–2024, prepared by the Ministry of Public Works in 2012 and approved by the Congress of Deputies in November 2013, is in line with the EU mobility policy, subordinating the future development of transport to sustainability and territorial cohesion. In it, parking is considered one of the key measures for strategic transport planning and must meet “(…) parking management criteria of a comprehensive and coordinated nature, in line with sustainable mobility policies (…)” (Ministerio de Fomento 2013:191). This shows that the environmental approach to mobility management is an important element of the PITVI 2012–2024.

**Fig. 4:**
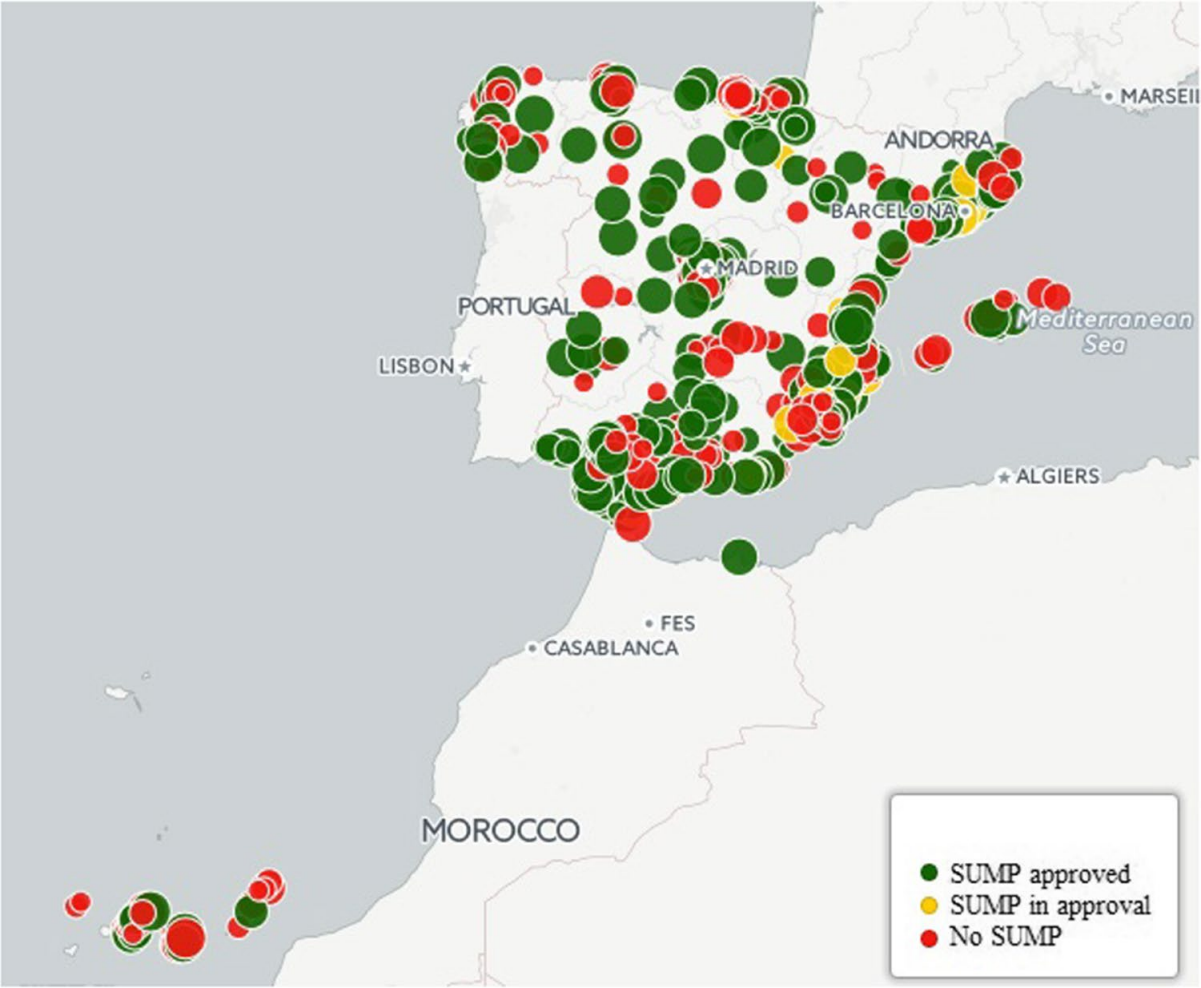
SUMPs in Spain in 2016. Source: own elaboration based on Ministerio para la transición ecológica (2021)

With regard to SUMPs, in 2006, the Ministry of Public Works and the Ministry of the Environment published a practical guide for the design and implementation of sustainable urban mobility plans, coordinated by the IDAE (Institute for Diversification and Saving of Energy of the Ministry of Industry) (Mozos-Blanco et al. 2018). This guide includes a section on new parking regulations that aim to limit the circulation of private vehicles through regulated surface parking, parking reserved for residents, or deterrent parking (Ministerio de Fomento 2006). In 2011, there was a decisive legislative change in the approval of SUMPs with the approval of Law 2/2011 on Sustainable Economy. This law defined the general principles and contents that all SUMPs had to include and, in addition, vetoed access to public transport funds financed by the public treasury for all those regions or municipalities that did not have an approved SUMP (BOE 2011a; Diez et al. 2018). The restriction to public funds meant that in 2014, the year in which Law 2/2011 came into force, 55 SUMPs were approved in Spain, whereas from 2005 to 2013, only 195 SUMPs were ratified. In 2016, the number of SUMPs approved or in the approval phase amounted to 295 plans (Fig. 4) (Davies Sala and Mínguez Alarcón 2016).

Besides guaranteeing access to public funds, the approval of SUMPs is necessary for the establishment of low emission zones (LEZs). With the approval of Law 7/2021 on Climate Change and Energy Transition, municipalities with more than 50,000 inhabitants, as well as island territories and municipalities with more than 20,000 inhabitants, that exceed the maximum levels of air pollution established in Royal Decree 102/2011, are required to implement a SUMP that establishes LZEs in the municipality. Thus, in Spain, LEZs are regulated within SUMPs. In this way, SUMPs are framed as holistic mobility strategies that create more efficient urban planning from a sustainability perspective. Although the Spanish municipalities that meet the above requirements represent only 1.8% of the total, they account for more than half of the Spanish population (56.5%), so the impact of this measure is high (MITECO 2021).

Regarding the validation of SUMPs, the most important cities in Spain have approved their implementation, such as Madrid (2014), Barcelona (2015), Valencia (2013), Seville (2019), Zaragoza (2006), Malaga (2012), Murcia (2013), Palma de Mallorca (2014), Bilbao (2018), Alicante (2013), Cordoba (2013), Valladolid (2015), Vitoria (2007), A Coruña (2014), and Granada (2013) (Fernández-González et al. 2022).

Despite the widespread adoption of SUMPs, the design of the plans and their implementation vary considerably. Focusing on Spain’s most populous cities, Madrid is quite deficient in terms of the lack of bicycle lanes and the limited scope of public awareness programs, but it has developed a public transport network that provides a viable alternative to private transport. Barcelona, for its part, has implemented policies in favor of pedestrian mobility and public transport, focusing mainly on the extension of bicycle lanes and a new orthogonal bus network. Nevertheless, the high use of private transport leads to high levels of noise, pollution, and accidents. Valencia is one of the cities where walking and cycling have increased the most, with the construction of a cycling network of more than 150 km. However, there has been resistance to change whenever space restrictions for private cars have been introduced. In the case of Seville, a new SUMP is needed to reinvigorate policies to promote high-capacity public transport (Garcia-Ayllon et al. 2021).

### The legal regime for sustainable mobility management

As discussed in the previous section, in the context of the European Strategy for Sustainable Mobility, Spain has two types of plans: the PITVI 2012–2024 and the SUMPs. These establish the guidelines for parking management as part of the sustainable mobility strategy. In addition to these plans, the institutional framework for the parking sector in Spain consists of two main laws, an Environmental Impact Assessment law and a law that regulates public sector contracts.

The first of these is Law 9/2006 on the Evaluation of the Environmental Impact of Certain Plans and Programs. The PITVI 2012–2024 and the SUMPs fall under the jurisdiction of this law because they are plans approved by the public administration, and, in addition, as stated in Article 3, Section a, these plans have significant effects on “(…) urban and rural land use or land use planning” (BOE 2006: 3). Therefore, according to the requirements of Article 7, Section a, it is essential to “prepare the environmental report” and to integrate “(…) an environmental sustainability report, the result of the consultations and the environmental report in the decision-making process” (BOE 2006: 4). From this law derives the need to evaluate the sustainability and the degree of respect for the ecosystem of each of the candidacies in the processes of awarding public parking contracts.

The second important law affecting the parking sector is Law 9/2017 on Public Contracts. This regulation transposes into the Spanish legal system the European Parliament and Council Directives 2014/23/EU and 2014/24/EU, approved in 2014. This law repeals Royal Legislative Decree 3/2011 and Law 30/2007, the two previous pieces of legislation that regulated public contracts. Both Law 30/2007 and Royal Legislative Decree 3/2011 and the current Law 9/2017 have two key points in common: the criterion of selecting the “most economically advantageous offer” and “compliance with environmental management” (BOE 2007, 2011b, 2017).

With regard to the most advantageous offer, each of these three legal systems specifies the importance of the winning offer also being the most profitable for the public purse (the criterion to be followed is to select the offer of equal or higher quality that offers a service at a lower price). Since 2007, with Law 30/2007, the concept of the “most economically advantageous offer” includes, in addition to financial criteria, “(…) mechanisms that allow the introduction of social and environmental considerations in public procurement, configuring them as special conditions for the execution of the contract or as criteria for the evaluation of the offers (…).” (BOE 2007: 8). This clarification highlights the importance of the environmental aspect in public tendering and is also specified in the article on environmental management standards in each of the legal regulations on public procurement. Law 9/2017, which is currently in force, includes in its Article 94 the obligation that “(…) contracting entities shall require, as a means of accrediting technical or professional solvency, the presentation of certificates issued by independent bodies accrediting that the candidate complies with certain environmental management standards (…)” (BOE 2017: 74) (Table 1).

**Table 1:** Environmental certifications of the most important Spanish companies in the parking sector

	Quality certificate UNE-EN ISO 9001		Environmental certificate UNE-EN ISO 14001		Energy performance certificate UNE-EN ISO 50001		Additional certificates
COMPANY	Certificate concession	Year of concession	Certificate concession	Year of concession	Certificate concession	Year of concession	
SABA APARCAMIENTOS S.A	Yes	2000	Yes	2008	Yes	2018	OHSAS 18,001 (occupational health and safety management)
EMPARK APARCAMIENTOS Y SERVICIOS S.A	Yes	2000	Yes	2008	No	-	
ESTACIONAMIENTOS Y SERVICIOS S.A	Yes	1997	Yes	2008	No	-	
DORNIER S.A	Yes	2000	Yes	2008	No	-	
INTERPARKING HISPANIA S.A	Yes	2000	Yes	2008	No	-	Carbon neutral certification (2014)
INDIGO INFRA ESPAÑA S.A.U	Yes	2005	Yes	2011	No	-	ISO 45001 (occupational health and safety management)
CONTINENTAL PARKING S.L	Yes	2014	Yes	2014	No	-	
APK80 APARCAMIENTOS S.L	Yes	n.a	No	-	No	-	
NEW CAPITAL 2000 S.L	Yes	2018	Yes	2018	No	-	
PAVAPARK MOVILIDAD S.L	Yes	> 2014	Yes	> 2014	Yes	2019	OHSAS 18,001 (occupational health and safety management) ISO 45001 (occupational health and safety management)
PARCLICK S.L	Yes	2005	Yes	2011	No	-	ISO 45001 (occupational health and safety management)
SERRANOPARK S.A	Yes	n.a	No	-	No	-	
TRANSANCHEZ S.L	Yes	n.a	No	-	No	-	
ISOLUX CORSAN APARCAMIENTOS S.A	Yes	2014	Yes	2014	No	-	ISO 45001 (occupational health and safety management)
GIROPARK S.A	Yes	n.a	No	-	No	-	

Source: own elaboration based on Continental Parking S.L. (2021), Empark (2016), ENAC Certification (2015), Indigo Infra España S.A.U. (2021), Interparking (2018), Isolux Corsán Aparcamientos S.A. (2021), New Capital 2000 S.L. (2021), Parclick S.L. (2020), Pavapark movilidad S.L. (2020), Saba infraestructuras, S.A. (2019)

## Methodology

The database used to obtain the data for the sample of this study is SABI (Iberian Balance Sheet Analysis System). SABI provides financial and economic information and other descriptive elements of 2,600,000 Spanish companies and 800,000 Portuguese companies over a period of 25 years (Ibarloza et al. 2018; SABI 2021).

In order to limit the population sample to the analysis sector of this article, the parking industry in Spain, and to the chosen time series, 2009–2019, the following search criteria were used: (1) the country in which the company’s tax address is located is Spain; (2) it must have an economic activity in at least one of the years between 2009 and 2019; (3) its CNAE code is 5221 (storage and ancillary transport activities); and (4) the word “parking” must be included in the description of its activity. After applying these four search filters, the resulting number of companies is 313.

In order to carry out a solid and complete analysis of this sector, two types of indices are used. The first group of indices is concentration indices, which refer to the market share of the companies. The second group is the stability indexes, which take into account the entry and exit trends of companies in this industry. The indexes have been calculated according to the formulas used in Fernández-González et al. (2021).

### The concentration indexes

1. Inverse number of entities (*R*)
  1 $$R=\frac{1}{N}$$

where,

*N*: number of companies in the sector.

2. Weight of the largest “*R*” entities ($CR$)where,
  2 $$CR=\sum_{i=1}^{k}{S}_{i}$$

where,

*R*: chosen number of the main market entities and ${S}_{i}$: market share of the -*i*th company (in order of highest to lowest).

3. Herfindahl–Hirschman index (HHI)
  3 $$\mathrm{HHI}=\sum_{i=1}^{N}{{S}_{i}}^{2}$$

where,

*N*: number of companies in the sector and ${S}_{i}$: market share of the -*i*th company (in order of highest to lowest).

4. Herfindahl–Hirschman standardized index (HHI-S)
  4 $$\mathrm{HHI}-\mathrm{S}=\frac{\mathrm{HHI}-\frac{1}{N}}{1-\frac{1}{N}}$$

where,

*N*: number of companies in the sector and $\mathrm{HHI}$: Herfindahl–Hirschman index.

5. Rosenbluth, Hall, and Tideman index (RHT)
  5 $$\mathrm{RHT}=\frac{1}{\left(2{\sum }_{i=1}^{N}i{S}_{i}\right)-1}$$

where,

*N*: number of companies in the sector; ${S}_{i}$: market share of the -*i*th company (in order of highest to lowest); and $i$: rank of the *i*-one entity in the industry.

6. Dominance index (DI)
  6 $$\mathrm{DI}=\sum_{i=1}^{N}{{\frac{{{S}^{2}}_{i}}{\mathrm{HHI}}}^{2}}_{i}$$

where,

*N*: number of companies in the sector; ${S}_{i}$: market share of the -*i*th company (in order of highest to lowest); and $HHI$: Herfindahl–Hirschman index.

### The stability indexes

7. Instability and volatility index
  7 $$I=\frac{1}{2}\sum_{i=1}^{N}\left|{S}_{i2}-{S}_{i1}\right|$$

where,

*N*: number of companies in the sector and ${S}_{i1}$ and ${S}_{i2}$: market share of the ith entity in periods 1 and 2, respectively.

8. Gross entry rate
  8 $${GEntryR}_{t}=\frac{{NE}_{t}}{{N}_{t-1}}$$

where,

*NE*_*t*_: number of companies entering the sector in the period *t* and ${N}_{t-1}$: number of companies in the sector in the period prior to *t*.

9. Gross exit rate
  9 $${GExitR}_{t}=\frac{{NL}_{t}}{{N}_{t-1}}$$

where,

*NL*_*t*_: number of companies leaving the sector in the period *t* and ${N}_{t-1}$: number of companies in the sector in the period prior to *t*.

10. Net entry rate
  10 $${NEntryR}_{t}=\left({{GEntryR}_{t}-GExitR}_{t}\right)\times 100$$

where,

*GEntryR*_*t*_: gross entry rate and ${GExitR}_{t}$: gross exit rate.

11. Market rotation rate
  11 $${RM}_{t}=\frac{{NE}_{t}+{NL}_{t}}{{N}_{t}}$$

where,

*NE*_*t*_: number of companies entering the sector in the period *t*.
*NL*_*t*_: number of companies leaving the sector in the period *t*.
*N*_*t*_: number of companies in the sector in the period *t*.

## Results

The values obtained from the calculation of the concentration and stability indices show a sector that, although not highly competitive, is not concentrated. The HHI index presents a value between 500 and 620 points throughout the period considered, which, according to the definition of the index itself, shows that the parking market in Spain is deconcentrated (Table 2). The HHI-S, the RHT, and the dominance index also show low values. In this sector, more than half of the market share is distributed among the top 10 companies and 80% of the market share belongs to the top 20 companies. Therefore, and taking into account the calculation of the indices, the value of the indices is low, since they are indices focused on monopoly and oligopoly and penalize the distribution of market power, even if it is among a not very large number of companies.

**Table 2:** Concentration indexes of the parking sector in Spain

	2019	2018	2017	2016	2015	2014	2013	2012	2011	2010	2009
Inverse number of entities index	0.00637	0.00565	0.00543	0.00529	0.00529	0.00526	0.00532	0.00562	0.00575	0.00602	0.00621
CR 1 index	15.77%	15.87%	11.54%	11.43%	12.38%	12.07%	12.76%	11.03%	10.61%	11.28%	14.01%
CR 3 index	35.05%	34.19%	30.62%	31.39%	32.30%	32.93%	34.83%	31.38%	29.86%	31.67%	35.12%
CR 5 index	48.99%	48.23%	45.32%	45.22%	46.52%	47.58%	49.39%	45.69%	45.08%	46.54%	48.76%
CR 10 index	66.50%	64.47%	62.41%	62.11%	62.58%	64.03%	65.65%	62.27%	63.03%	63.91%	66.32%
HHI index	619.31	598.65	511.52	515.49	537.47	561.90	604.48	530.33	516.97	553.85	613.00
HHI standardized index	0.0616	0.0596	0.0508	0.0512	0.0535	0.0559	0.0602	0.0527	0.0514	0.0551	0.0610
RHT index	0.0354	0.0314	0.0286	0.0278	0.0294	0.0292	0.0313	0.0312	0.0319	0.0317	0.0327
Dominance index	0.2247	0.2374	0.1662	0.1665	0.1780	0.1753	0.1824	0.1611	0.1429	0.1575	0.1886

However, in all the indices, an increase in their values can be observed in 2018 and 2019. In these years, the three leading companies in the market have intensified their acquisitions of other companies in the sector. Since *2014*, *Saba aparcamien*tos S.A. has absorbed 7 companies, *Empark aparcamientos y servicios S.*A. has become the sole shareholder of 15 companies, and *Estacionamientos y Servicios S.A*. has bought 9 companies in the sector, with 40% of these transactions occurring in 2018 and 2019 (SABI 2021).

**Fig. 5:**
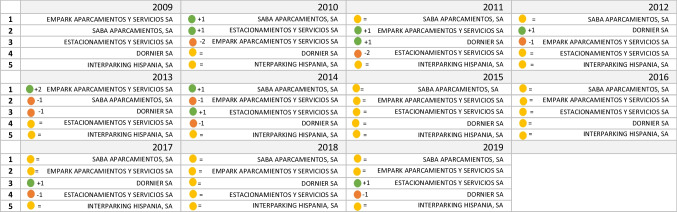
Top five companies with the largest market share in the parking sector in Spain

As shown in Fig. 5, throughout the study period, the five dominant companies are the same (*Saba Aparcamientos S.A*., *Estacionamientos y Servicios S.A.*, *Empark Aparcamientos y Servicios S.A*., *Dornier S.A*., and *Interparking Hispania S.A*.). In these 11 years, there are no new entries in the top 5 companies of the sector, which shows a consolidation of the market shares of these companies, especially since 2015. In fact, the change in the positions of the top 5 companies is minimal in the last 5 years. The first, second, and last positions have not changed. This immobility is due to the exploitation of economies of scale by the largest companies in the sector, the policy of takeovers, and the high number of public concessions due, among other things, to the possession of environmental certificates that increase the valuation of companies in public tenders.

This situation has led to the existence of a dichotomous scenario in the Spanish parking sector. During the period of analysis of this study, the top 15 companies are large and medium companies and the remaining companies, more than 250, are small and micro companies. The companies in the sector offer a very homogeneous product, the parking management service, but in public tenders the large companies offer a more competitive bid, which allows them to win the public contract and consolidate their position in the market.

In addition to the level of concentration, which has already been analyzed, it is also important to know the level of stability of the sector. The evolution of the instability and volatility index shows a market that, with the exception of 2019, has not shown major fluctuations. Between 2009 and 2013, a period in which the economic crisis had the most serious effects in Spain and in which the implementation of Law 9/2006, Law 30/2007, and Law 2/2011, which affected the environmental aspect of parking management, was recent, there were no high volatility values in the market.

Between 2009 and 2019, 75 companies entered the market and 77 companies left it, resulting in an almost unchanged number of companies in the market. However, two distinct phases with different trends can be observed.

Between 2009 and 2014, the market rotation rate is positive (Table 3). This is because, in the face of the economic crisis in Spain, the parking sector represented a safe haven market, where small investors came and created small and micro companies. This trend occurred regardless of the fact that the parking sector in Spain was going through a period of stagnation at the time. Until 2014, the sector suffered a decline in net revenues of between 0.8% and 3.4%. The economic crisis, the reduction in demand, and the lower number of municipal concessions were the reasons for the decline in the sector’s revenues (DBK 2011). Nevertheless, the parking sector offered more opportunities for investors than other markets.

**Table 3:** Stability indexes of the parking sector in Spain

	2019	2018	2017	2016	2015	2014	2013	2012	2011	2010	2009
Instability and volatility index	0.1401	0.0395	0.048	0.0529	0.0582	0.0736	0.0851	0.0898	0.1034	0.0903	0.0869
Gross entry rate	0.56%	0.00%	1.06%	2.65%	2.63%	4.26%	7.30%	5.75%	7.83%	6.21%	5.06%
Gross exit rate	11.86%	3.80%	3.70%	2.65%	3.16%	3.19%	1.69%	3.45%	3.01%	3.11%	3.80%
Net entry rate	0.56%	0.00%	1.06%	2.65%	2.63%	4.26%	7.30%	5.75%	7.83%	6.21%	5.06%
Market rotation rate	− 11.30%	− 3.80%	− 2.65%	− 0.01%	− 0.53%	1.06%	5.62%	2.30%	4.82%	3.11%	1.27%

The second phase of the sector begins in 2015, when the market turnover rate shows negative values. With the economic recovery and the increase in the number of road trips came the expansion of surface regulated parking zones in small- and medium-sized cities (DBK 2014). This type of parking was the driving force behind the growth of the sector, although the number of parking structures also increased as the construction sector was reactivated and central locations became profitable due to the increase in demand (DBK 2021). The improvement in the macroeconomic situation in Spain also led small investors to focus their attention on other markets. In addition, this factor was complemented by the expansion policy of the large companies in the market. The dominant companies intensified their acquisition strategy, being particularly aggressive in 2019.

The sector’s recovery was largely based on new public parking concessions. Large companies in the sector strengthened their position in the market by gaining market share through winning public tenders, partly due to the possession of environmental certificates, which gave them a comparative advantage. The low competitiveness of small and micro enterprises in public parking tenders forced some of them to withdraw from the market. In addition, the acquisition policy of the dominant companies has led to a high gross exit rate since 2014.

## Conclusions

Laws and regulations play a fundamental role in economic allocation. As a result of the legal framework promoted by Law 30/2007, since 2007. public contracts have included environmental criteria in the selection of the company to be awarded the contract, as well as in the evaluation of the purely economic part of the offer. It is obvious that large companies in the market, due to economies of scale and market position, can offer a comparative economic advantage over small- and medium-sized companies in the sector. However, in addition to these factors, by including strict environmental criteria in public tenders and requiring compliance through certification, large companies have also taken advantage of this requirement to strengthen and increase their market position.

Law 30/2007, Royal Legislative Decree 3/2011, and the current Law 9/2017 require the presentation of UNE-EN ISO 9001 and UNE-EN ISO 14001 certificates to accredit the technical and environmental solvency of companies (Tribunal de Contratación de Madrid 2015). As shown in Table 1, the main companies in the market hold these certificates and even add to their portfolio new certificates related to the sustainable management of their activities. In fact, the dominant companies were the first to hold these certificates in the sector, which has given them a comparative advantage.

The institutional framework created by the PITVI 2012–2024 and Law 2/2011 on the Sustainable Economy, which made transport funds conditional on the approval of a SUMP, has led public decision-makers to attach greater importance to environmental issues in the tendering process. This tendency to emphasize environmental aspects is gaining momentum, so it seems that those companies that are committed to green management will be better positioned in the market.

According to the analysis conducted from 2009 to 2017, the results of the concentration indices reflect that the market is competitive but not highly concentrated. The HHI index, one of the reference indices, shows values close to 550 points, indicating that there is no high market power during this period of the study. Thus, in the last 2 years analyzed in this study, the dominant companies have increased their market share. While it is certain that their economic and financial position and acquisition policy have been relevant factors in explaining this process, it is also evident that their firm commitment to environmental certification has been a determining factor.

The increasing concentration in the parking market is a phenomenon that is also reflected in the stability indices analyzed in this case study. While from 2009 to 2014, the market rotation rate was positive, indicating that this sector was attracting new companies; since 2015, this rate has been negative. Thus, there is a tendency for this sector to be composed of fewer companies. This scenario is in line with the tightening of environmental conditions for access to public contracts, the approval of new regulations on sustainable urban mobility, and the increase in the approval of SUMPs.

Despite the fact that the market shows an acceptable level of competitiveness in terms of concentration indices and that the turnover ratios of this market show fluctuations compatible with an unrestricted market, the leadership of the sector remains practically static. In the period 2009–2019, 75 companies entered the market, while 77 others left it, and in addition, the entry of companies whose main activity was civil engineering, but who were looking for a secondary business in the parking management sector, was consolidated. However, these factors have not led to a significant change in the top 5 leading companies in the market. Companies such as *Empark Aparcamientos y Servicios S.A.*, *Saba Aparcamiento S.A*., *Dornier S.A.*, or *Estacionamientos y Servicios S.A*. have dominated the market over the 11 years analyzed, with minor changes in the leading positions. All of these companies were pioneers in obtaining environmental certification.

Therefore, environmental policies and their stricter compliance will be a determining factor for the profitability of these companies. At the same time, the construction of park-and-ride lots is expected to increase due to restrictions on private vehicle mobility. Future lines of research will deal with this new scenario to find out how it will affect the companies in the sector financially. This future analysis will overcome the limitations present in this article by incorporating new factors into the analysis, such as the allocation of public contracts.

## Data Availability

The data that support the findings of this study are available from the corresponding author, R F-G, upon reasonable request.
